# Iatrogenic Cushing’s From Celiac Plexus Blocks for Chronic Pancreatitis: A Case Report

**DOI:** 10.7759/cureus.34703

**Published:** 2023-02-06

**Authors:** Ariana R Tagliaferri, Sewar H Abuarqob, Yana Cavanagh

**Affiliations:** 1 Internal Medicine, St. Joseph's Regional Medical Center, Paterson, USA; 2 Internal Medicine, St. Joseph's University Medical Center, Paterson, USA; 3 Gastroenterology, St. Joseph's Regional Medical Center, Paterson, USA

**Keywords:** abdominal pain, nerve block, celiac plexus, pancreatitis chronic, cushing’s syndrome, iatrogenic disease

## Abstract

Chronic pancreatitis and pancreatic malignancies can result in chronic pain that is difficult to treat with traditional regimens. Various pain management strategies have been implemented to improve the quality of life for patients with these conditions, but these strategies are limited by their efficacy and side effects, including opiate dependence. Celiac plexus blocks (CPB) and celiac plexus neurolysis (CPN) were implemented to decrease opiate dependency and treat chronic pain for pancreatitis and pancreatic malignancy. Numerous approaches are used to facilitate CPB/CPN, including percutaneous, surgical, and endoscopic, guided as computerized tomography (CT), fluoroscopy, ultrasound (US), or endoscopic ultrasound (EUS) techniques. EUS is the latest development in CPB/CPN and the least commonly utilized method; however, it is highly efficacious and associated with minimal complications and/or risks. With endoscopic CPB/CPN, overall mortality improves. Despite the various complications associated with other techniques, no case report or current literature has documented the development of iatrogenic Cushing’s disease from the use of steroids during CPB via any approach. Herein, we report the first case of iatrogenic Cushing’s disease from CPB in the treatment of chronic pancreatitis. Future studies are warranted to examine the agents used in the chemical destruction for CPB/CPN, to avoid complications such as this.

## Introduction

Chronic abdominal pain can be a burden for patients with underlying malignancies and/or benign diseases [1,2]. Various pain management strategies are implemented to improve the quality of life but are limited by their efficacy and side effects [1,2]. Nociceptive pain is a type of pain caused by injury to either superficial tissue, muscle, or bone, also known as somatic pain. Injury to the organs directly is known as visceral pain [1]. When there is injury to the nerves, it is called neuropathic pain [1]. When there is a combination of these classifications, it is called neurotropic pain [1]. Those with the pancreatic disease typically have neurotropic pain and are thus difficult to treat [1]. Although the pain is multifactorial, pancreatic pain is likely from celiac plexus invasion by tumor infiltration, pancreatic duct obstruction and distention, inflammation, and/or ischemia [3]. Opiates and non-steroidal anti-inflammatory drugs (NSAIDs) have historically been used in the treatment of neurotropic pain, but they are often ineffective and lead to side effects such as constipation, emesis, and sedation, and can result in addiction [1,4,5]. Additionally, studies have shown that chronic pain increases the overall risk of morbidity and mortality [1]. The celiac plexus innervates the upper abdominal viscera. A celiac plexus block (CPB) is a minimally invasive technique performed in various ways to decrease neurotropic pain in those with chronic upper abdominal diseases such as pancreatic or gastric malignancies and/or chronic pancreatitis resulting in pain [1]. The strategy of CPB and celiac plexus neurolysis (CPN) is direct intervention at the level of the celiac plexus, inhibiting any downstream transmission of pain signaling in the upper abdomen [1]. Celiac interventions are performed based on anatomical landmarks, but now modern practices include percutaneous, endoscopic, surgical, or computerized tomography (CT)-guided interventions [4]. Through the use of a multi-modal approach incorporating CPB, opiate dependence decreases and overall mortality and baseline functioning improves [1,2,4,5]. 

CPB was first described in 1914 by a surgical anesthesiologist who wanted to chemically destroy the nerve fibers transmitting somatic and visceral pain in the abdomen [4,6]. By 1957, CPB was used as an adjunctive measure to treat pain in cancer patients [4]. CPN is the chemical destruction of the neural ganglion, which is better indicated for those with cancer pain [3]. About 50-70% of patients with pancreatic cancer have chronic pain unresponsive to opiates, and thus CPN is an effective method with a small side effect profile and confers little risk to the patient [6]. In comparison to CPN, CPB chemically inhibits the ganglion, temporarily impairing neuronal transmission. This method is more appropriate for those with chronic pain from pancreatitis who have failed other pain management approaches [3,6]. A study comparing the efficacy of CPN in chronic pancreatitis versus pancreatic cancer concluded that those with pancreatic cancer not only had complete relief of pain initially but also a longer duration of treatment, experiencing little to no pain until death [3]. Conversely, those with pancreatitis had lower rates of complete initial remission and shorter durations until symptom recurrence [3]. Many studies have shown adequate pain control with CPB in chronic pancreatitis; however, more randomized controlled trials are warranted to compare techniques and overall outcomes [2,4].

This article was previously presented as an abstract at the Annual ACG Conference in North Carolina, October 2022.

## Case presentation

A 27-year-old female with a past medical history of chronic pancreatitis, irritable bowel syndrome, gastroparesis, endometriosis, and bipolar disorder presented to the emergency department (ED) with complaints of severe epigastric pain associated with nausea. The pain was rated a 10/10 on the numerical rating scale. The patient had a history of chronic pancreatitis, diagnosed a year and a half prior. At the time of diagnosis, she underwent an endoscopic ultrasound (EUS) with biopsies positive for benign pancreatic tissue, fibrotic stromal tissue, and scattered eosinophilic infiltrates. The underlying cause of chronic recurrent pancreatitis was idiopathic and non-hereditary. A cystic fibrosis transmembrane conductance regulator (CFTR) and PRSS1 gene analysis, as well as an autoimmune workup, were negative. Additionally, triglycerides were always less than levels of 400 mg/dL. 

When she presented to the ED, her other medical history was significant for bipolar disorder with depressive features, for which she took quetiapine extended-release tablet 300 milligrams daily, irritable bowel syndrome, and endometriosis, for which she underwent a laparoscopy 10 years prior. For her chronic pain, the patient was also taking ibuprofen 400 milligrams every six hours standing, famotidine 40 milligrams daily, oxycodone 10 milligrams every four hours as needed for pain, and acetaminophen 650 milligrams every six hours standing. She had no known allergies to medications, was a lifetime non-smoker and did not use alcohol or illicit drugs. There was no family history of pancreatitis, autoimmune diseases, or malignancy. 

**Figure 1:**
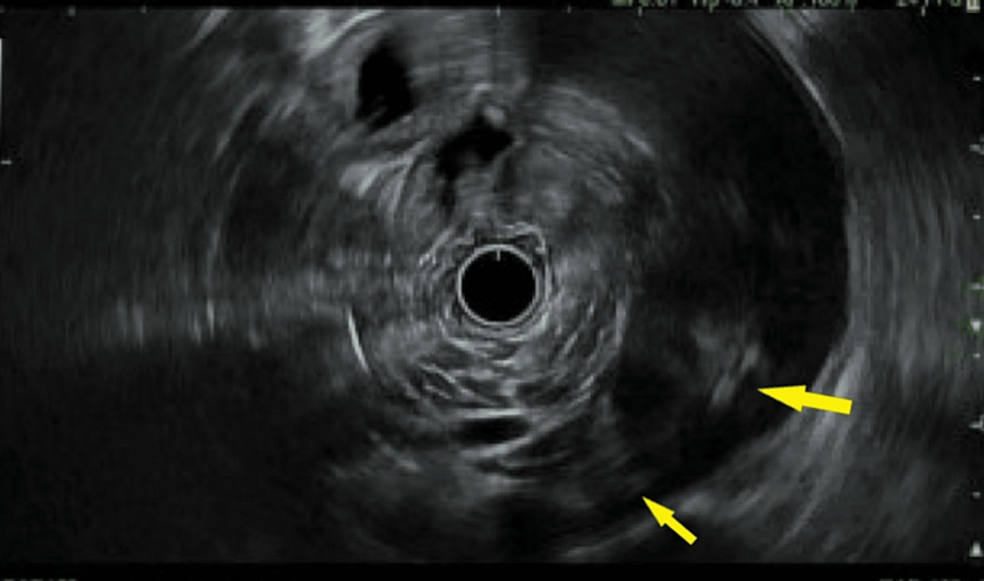
Initial EUS of the pancreas demonstrating moderate-to-severe chronic pancreatitis. Shown is the initial EUS demonstrating moderate-to-severe chronic pancreatitis. Arrows indicate chronic calcifications and heterogeneity of the pancreas. EUS: endoscopic ultrasound.

In the ED, her recurrent pain was secondary to pancreatitis, which was confirmed by an EUS demonstrating gastritis and moderate-to-severe chronic pancreatitis (Figure 1). 

**Figure 2:**
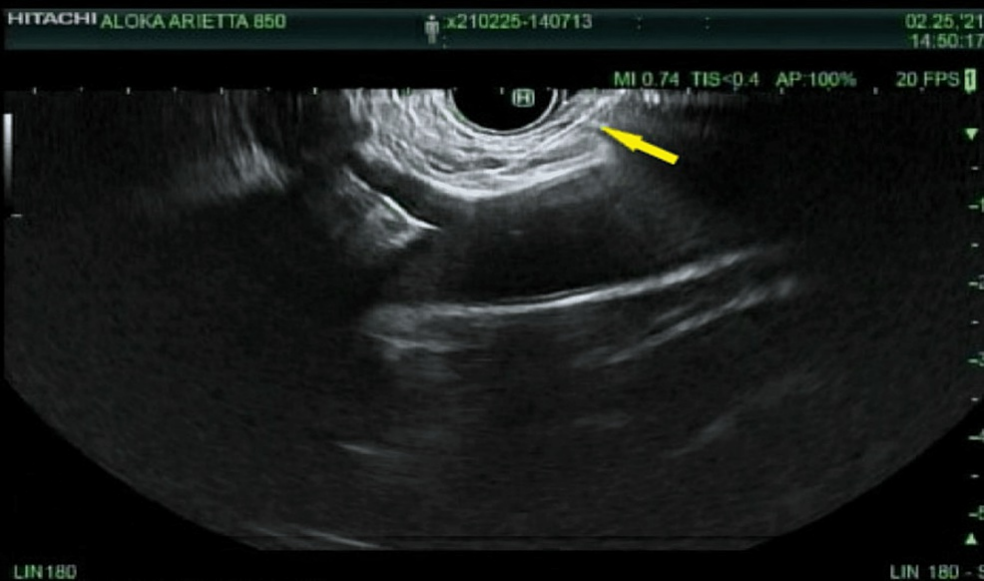
EUS-guided celiac plexus nerve block. Shown is the EUS-guided celiac plexus nerve block. The arrow indicates a 22-gauge needle inserted into the celiac ganglia. EUS: endoscopic ultrasound.

She was discharged and referred to a pain management specialist within one month. Hydromorphone, 4 milligrams every three hours as needed, and methadone, 30 milligrams twice daily, were added to her pain regimen. She was also maintained on pancrelipase 10,000 units three times daily. One month later, she had refractory pain upon follow-up to the gastroenterology clinic, (8-10/10 on the numerical rating scale) despite an optimal regimen. For this, she underwent a celiac plexus block via EUS, which also demonstrated honeycombing in the entire pancreas, suggestive of chronic pancreatitis and a 1-centimeter (cm) hiatal hernia (Figure 2).

The celiac plexus block was performed using a trans-gastric approach, in which a 22-gauge needle was advanced to the area of the celiac ganglia. A total of 20 milliliters using 0.25% bupivacaine and 80 milligrams of triamcinolone (40 milligrams per milliliter) were injected into the celiac plexus block. After one celiac plexus nerve block, the pain was unresolved upon one-month follow-up, at which time she reported an 8/10 on the numerical rating scale. She was started on oxycodone-acetaminophen 10/325 milligrams every four hours as needed, and a fentanyl patch 75 micrograms per hour every 72 hours, by pain management. A subsequent nerve block was repeated four months later with complete resolution of pain. The dosing was the same as the first procedure.

During this time period, the patient also had a gastric emptying study due to gastroparesis showing delayed gastric emptying, for which she was treated with a gastric peroral endoscopic myotomy (G-POEM) one month after her second nerve block. After a total of two months following her last celiac block, another was repeated using 30 milliliters of 0.5% bupivacaine and 200 milligrams of triamcinolone (40 milligrams per milliliter). Subsequently, she presented to the ED within 30 days for recurrent 10/10 pain, requiring a hydromorphone pump and repeat celiac plexus block using the same dosing as the previous procedure. She was discharged with a numerical rating scale of 0/10; however, this only lasted for two months, when she reported a return of 10/10 abdominal pain in the gastroenterology clinic. At that visit, she also endorsed a weight gain of ten pounds, fatigue, increased acne and facial hair, facial fullness, dark purple abdominal striae, and insomnia. 

Through cortisol testing and the symptoms described, the patient was suspected to have resultant iatrogenic cushingoid features due to multiple (four) celiac nerve blocks. A morning cortisol level was 0.3 μg/dL, but three late-night salivary cortisol levels were within normal limits (<0.010 μg/dL). These were drawn on three consecutive days. A morning adrenocorticotropic hormone (ACTH) was also drawn at this time, and the results demonstrated low levels (6 pg/mL). A 1 mg dexamethasone suppression test was administered overnight with morning cortisol results again at 0.3 μg/dL. She was referred to endocrinology, who recommended withholding steroids for at least three to six months. At this stage, it was likely that exogenous steroid use had caused secondary adrenal insufficiency. After approximately five months without steroids, her Cushingoid symptoms resolved, but the pain returned and her methadone was increased to 50 milligrams every twelve hours.

She underwent esophagogastroduodenoscopy (EGD) and EUS, which showed gastritis with erythematous mucosa in the gastric cardia and pancreatic parenchymal abnormalities consisting of hyperechoic strands, hyperechoic foci, and lobularity throughout the entire pancreas, again consistent with chronic pancreatitis. Due to refractory pain, she was referred to a pancreatic transplant program. In the interim, she continued to undergo EUS-guided celiac plexus blocks to treat her pain with the same agents. 

## Discussion

The celiac plexus is located in the retroperitoneum at the level of the celiac and superior mesenteric arteries, and contains the celiac, superior mesenteric, and aorticorenal ganglia [1,4]. The celiac plexus transmits parasympathetic and sympathetic signaling to the pancreas, liver, spleen, biliary tract, gallbladder, adrenals, kidneys, mesentery, stomach, and proximal transverse colon [1,4]. There are numerous celiac ganglia, present in the fat pads posterior to the stomach and pancreas, corresponding to the levels between thoracic (T) 12 and lumbar (L) 1 [1,4]. In 1919, the first CPB retrocrural technique was described, in which the patient lies in the lateral decubitus position and the T12 and L1 vertebral landmarks are used to blindly insert a needle at the lateral border of the paraspinal muscle along the lower border of the 12th rib [4,6]. This method effectively blocks the splanchnic innervation; however, by 1978, the blind transcrural approach was introduced to block the celiac plexus fibers along the anterolateral path with the patient lying in the prone position [4,6]. Other approaches include the transaortic method, where the patient lies in a prone position and the needle passes anterior to the aorta and diaphragmatic crus, and the trans-discal approach, where the patient is also prone and the needle is passed through the intervertebral disc space [4]. Any method can be performed using CT scans, magnetic resonance imaging (MRI), fluoroscopy, ultrasound, or EUS for guidance [4]. One of the first studies on EUS-guided CPB in pancreatitis was in 1996, it remains underutilized [1,3]. Today, CT-guided CPB is the preferred method due to its cross-sectional nature, although there is no gold standard technique or modality worldwide [1,4]. CPB can be performed intraoperatively during abdominal surgeries as well [6]. Recent studies have demonstrated that EUS-guided CPB carries lower complication rates than percutaneous approaches due to the use of doppler and an anterior approach, although no studies have shown significant differences in terms of pain relief or overall outcomes compared to percutaneous approaches [1,6,7].

Typically, anesthetics such as bupivacaine and epinephrine mixtures are used in CPB [1,3]. Often steroids are added to this mixture when treating chronic pancreatitis [5]. Ethanol or phenol is used for CPN at concentrations greater than 50% [1,3]. Contraindications to CPB or CPN include coagulopathies, bowel obstruction, abdominal infections, and history of abdominal aneurysms [5]. Serious complications occur in less than 2% of patients [1,3]. Although many patients may experience transient back pain following the procedure, other common adverse effects can include diarrhea or orthostatic hypotension in up to 40% of patients [1,6]. Paresthesia is the most severe but very rare adverse effect, accounting for less than 1% of all complications [1,5]. To date, no case reports have documented adverse effects from steroid injections during CPB [2].

It is likely that her chronic pain from pancreatitis was also confounded by her underlying depression and irritable bowel syndrome; however, the use of opioids, anti-inflammatory medications, patches, and frequent follow-up intervals did not resolve the pain. The only treatment that temporarily relieved her pain was the CPB, performed endoscopically. She had no complications post-operatively, however, underwent four celiac plexus nerve blocks at increasing doses of steroid administration prior to developing any symptoms related to Cushing's syndrome. The diagnosis of Cushing's syndrome and secondary adrenal insufficiency also provided unique challenges, as previous studies have shown that adrenal insufficiency is secondary to chronic opioid use [8]. Both opioids and steroids suppress the hypothalamic-pituitary axis causing decreased ACTH levels and cortisol; however, the treatment for Cushing's syndrome is a withdrawal of the offending agent, whereas the treatment of opioid-induced adrenal insufficiency is glucocorticoid replacement [8]. About 9 to 29% of patients using opioids can develop this complication, but the timeline in which this occurs is unknown [8]. Because the patient was on opioids for almost two years prior to the initiation of CPB using steroids, it was inferred that this was the cause of her adrenal insufficiency and Cushingoid features. 

## Conclusions

Our patient with chronic pancreatitis was treated with EUS-guided CPB with the use of steroid-containing solutions. Although her pain was relieved with CPB, she developed iatrogenic Cushing’s disease. To our knowledge, this has not been previously reported in the current literature. Due to this complication, the patient warrants referral to a pancreatic transplant center. EUS-guided CPB is an effective, minimally invasive modality in treating pain from abdominal malignancy and/or pancreatitis; however, it is imperative that future studies evaluate the agents used for chemical destruction to avoid complications such as iatrogenic Cushing’s disease.
